# Carbonic anhydrase IX and response to postmastectomy radiotherapy in high-risk breast cancer: a subgroup analysis of the DBCG82 b and c trials

**DOI:** 10.1186/bcr1981

**Published:** 2008-03-20

**Authors:** Marianne Kyndi, Flemming B Sørensen, Helle Knudsen, Jan Alsner, Marie Overgaard, Hanne M Nielsen, Jens Overgaard

**Affiliations:** 1 Department of Experimental Clinical Oncology, Århus University Hospital, Nørrebrogade, 8000 Århus C, Denmark; 2 Department of Pathology, Århus University Hospital, Nørrebrogade, 8000 Århus C, Denmark; 3 Department of Pathology, Herlev Hospital, Herlev Ringvej, 2730 Herlev, Denmark; 4 Department of Oncology, Århus University Hospital, Nørrebrogade, 8000 Århus C, Denmark

## Abstract

**Introduction:**

A significant survival improvement after postmastectomy radiotherapy was identified in the Danish Breast Cancer Cooperative Group (DBCG82) b and c studies and in the British Columbia Randomized Radiation Trial. Recently, potential predictive value regarding response to postmastectomy radiotherapy was reported for carbonic anhydrase (CA) IX in a study (reported in abstract form) that included 160 patients. The purpose of the present study was to examine the importance of CA IX to response to postmastectomy radiotherapy in the larger scaled DBCG82 b and c studies.

**Methods:**

The DBCG82 b and c studies included 3,083 high-risk Danish breast cancer patients. The women were randomly assigned to postmastectomy radiotherapy plus systemic therapy (cyclophosfamide, methotrexate and fluorouracil in premenopausal women; and tamoxifen in postmenopausal women) or to systemic therapy alone. Cores from invasive tumour-containing paraffin blocks from 1,000 patients (more than seven nodes surgically removed) were transferred to tissue microarrays. Tissue microarray sections were stained immunohistochemically for CA IX (M75). The median follow up for patients remaining alive was 17 years. Clinical end-points were loco-regional recurrence, distant metastases, disease-specific survival and overall survival. Statistical analyses included κ statistics, χ^2 ^or exact tests, Kaplan-Meier probability plots, Log-rank test and Cox regression analyses.

**Results:**

CA IX was assessable in 945 cores. The percentage of tumours positive for CA IX was 16% (≥ 10% invasive tumour staining). CA IX was not an independent prognostic marker for survival, distant metastases, or locoregional recurrence in the subgroup of 945 patients or within either of the two randomization arms. In subgroup analyses, however, CA IX was an independent prognostic marker for overall survival among postmenopausal women (*P *= 0.001), women with one to three positive nodes (*P *= 0.02) and hormone receptor positive women (*P *= 0.001). Fifteen-year probabilities of overall survival were improved by 9% and 7% after postmastectomy radiotherapy for the subgroups of CA IX negative and CA IX positive patients, respectively.

**Conclusion:**

Within this series of 945 high-risk premenopausal and postmenopausal women, positivity for CA IX was not overall an independent prognostic marker for survival; only in subgroup analyses was it found to have prognostic value. The improvement in 15-year survival after postmastectomy radiotherapy was of similar magnitude in the two subgroups of CA IX positive and CA IX negative patients.

## Introduction

The zink metalloenzyme carbonic anhydrase (CA) IX is a transmembrane glycoprotein that reversibly converts carbon dioxide and water to carbonic acid. CA IX has been shown to play an important role in pH regulation and its expression has been suggested, in response to hypoxia, to reduce the pericellular pH and facilitating breakdown of the extracellular matrix [1]. In addition, it may play a role in cell proliferation and cellular transformation [2] as well as in adaptation of tumour cells to hypoxic conditions [3-5]. It is well known that the response of cells to radiation is dependent on oxygen [6], and consequently CA IX has been linked to response to radiotherapy [7-9].

CA IX over-expression in breast cancer has been associated with over-expression of human epidermal growth factor (HER)2 [10] and with reduced survival [11,12]; Cox multivariate regression analyses have revealed these associations to be statistically significant [7,8,13,14]. In the largest study conducted to date in this area (including 400 patients), the prognostic effect of CA IX was restricted to premenopausal women also treated with tamoxifen and to premenopausal women not treated with tamoxifen and with one to three positive lymph nodes [8]. Additionally, the gene encoding CA IX was among the 231 that were most upregulated or downregulated in the studies conducted by Van't Veer and coworkers [15,16], in which the 70-gene signature was discovered.

Chia and coworkers [7] recently examined the predictive value of CA IX in the 160 out of 318 patients who were randomly assigned to postmastectomy radiotherapy (PMRT). By applying immunohistochemical analyses to tissue microarrays (TMAs), the investigators found that 23% of tumours were positive for CA IX (with at least one invasive tumour cell staining being deemed to represent positivity). Positivity for CA IX was an independent prognostic marker for reduced survival. Furthermore, significantly improved survival after PMRT was found among CA IX positive patients but not in the larger group of CA IX negative patients. These results are in disagreement with the theory that aggressive tumours (characterized by independent prognostic markers of reduced survival) will, at an early time point in the pathogenesis, seed tumour cells to distant sites where they can develop into distant micrometastases. If these distant micrometastases additionally were resistant to the systemic therapy applied, then PMRT – which eliminates tumour cells in the radiated area only – would not result in improved survival for this poor prognosis group. Furthermore, the findings are in disagreement with the theory that CA IX positive status is a hypoxic marker and is associated with increased radio-resistance, and is therefore associated with reduced improvement in locoregional recurrence (LRR) control and subsequently survival after PMRT.

We should like to test three hypotheses. First, we hypothesize that positive CA IX status is an independent prognostic marker for increased invasive potential, and thereby is associated with increased probability of LRR, distant metastases (DM), and reduced survival. Second, we hypothesize, counter to the findings presented thus far, that if positive CA IX status is an independent marker of reduced survival then an improvement in survival after PMRT will be restricted to CA IX negative patients. Finally, we hypothesize that CA IX positive patients experience a smaller reduction in LRR after PMRT as compared with CA IX negative patients.

## Materials and methods

### Patients

Twenty-seven patients with at least two paraffin blocks from the same primary tumour were selected from a cohort described previously [17]. They were included in a pilot study comparing staining of TMA biopsies with staining of whole sections.

In addition, a subgroup, extracted from among the 3,083 high-risk Danish breast cancer patients included in the Danish Breast Cancer Cooperative Group (DBCG82) b and c studies in the period from 1982 to 1989, was selected for biological analyses. The 3,083 DBCG82 patients have been described previously [18,19]. In brief, high risk was defined as either positive lymph nodes and/or tumour size larger than 5 cm and/or invasion of tumour to surrounding skin or pectoral fascia. All women had undergone a total mastectomy and a partial axillary dissection. The premenopausal women were enrolled in the DBCG82 b protocol and were randomly assigned to receive either radiotherapy plus CMF (cyclophosfamide, methotrexate and fluorouracil) chemotherapy (eight cycles) or to CMF chemotherapy alone (nine cycles) [18]. The postmenopausal women were enrolled in the DBCG82 c protocol and were randomly assigned to either radiotherapy plus tamoxifen (30 mg daily/1 year) or to tamoxifen alone [19]. Recently, long-term clinical follow up in these patients was reported [20]. Median follow-up time was 17 years for patients remaining alive. The subgroup, selected for biological analyses, included 1,078 patients; for all of these patients paraffin blocks were available and more than seven lymph nodes were surgically removed (median seven lymph nodes removed).

## Methods

At least two paraffin-embedded tumour blocks from each of the 27 patients included in the pilot study and at least one from each of 1,078 DBCG82 b and c patients were collected, sectioned, and stained with haematoxylin and eosin. Invasive tumour was verified and marked on all 54 paraffin blocks from the pilot study and on 1,000 out of the 1,078 DBCG82 paraffin blocks. A central core and a peripheral 1 mm TMA core punched from the area previously identified as including invasive tumour were transferred to TMAs from each of the 54 paraffin blocks. Only a central 1 mm core from each of the 1,000 DBCG82 paraffin blocks was transferred to TMAs. All TMAs and the 54 paraffin blocks were sectioned, and sections were stored at 4°C until manual staining for CA IX.

Handling of the biological material was previously described in detail, including TMA construction and immunohistochemical staining for oestrogen receptor (ER) and progesterone receptor (PgR), and immunohistochemical staining and fluoresence *in situ *hybridization analyses for the HER2 receptor [21]. Before immunohistochemical staining for CA IX, de-paraffinization with 99% ethanol, blocking of the endogen peroxidase with H_2_O_2_, and epitope retrieval by microwave was performed. Sections were immunohistochemically stained for CA IX with the monoclonal antibody M75 at a dilution of 1:2,500. The ability of this antibody to detect CA IX expression specifically in tissue sections was previously confirmed by direct correlation with Western blot analysis in human breast tumour specimens [22].

The sections were incubated overnight at a temperature of 4°C. On day 2 the sections were incubated with a peroxidase conjugated to goat antimouse immunoglobulins (DAKO Envision Mouse, K4001; Dako, Glostrup, Denmark) for 60 minutes at room temperature. Afterward, sections were stained with DAB (K3468; Dako) and counterstained with haematoxylin. Sections from a control TMA, including invasive breast tumour cores previously determined to be CA IX positive and negative, were stained with and without primary antibody as well. All sections from the pilot study were scored semiquantitatively by two observers, and sections from the DBCG82 b and c studies were scored semiquantitatively by one observer. If there was any doubt regarding the scoring of the DBCG82 b and c sections, then a second observer was consulted and consensus was reached on the diagnosis. Percentage of tumours with membrane staining for CA IX was recorded. In the DBCG82 b and c studies membrane staining in at least 10% of invasive tumour was scored as positive.

### Statistical analyses and end-points

The pilot study was analyzed using κ statistics. In the DBCG82 b and c studies χ^2 ^or exact tests were used to test relationships between variables. Kaplan-Meier survival probability curves were constructed and evaluated for differences using a log-rank test. The prognostic value of CA IX was analyzed using Cox univariate and multivariate regression analyses. Tests of interactions were applied to Cox multivariate regression analyses as well. *P *< 0.05 was regarded to indicate statistical significance, and all estimated *P *values were two tailed. Statistical end-points were isolated LRR, DM, disease-specific survival (DSS) and overall survival (OS). Hazard ratios (HRs) provided on Kaplan-Meier OS probability plots were HRs of overall mortality. Statistical calculations were performed using the statistical program STATA version 8.2 (Stat Corp., College Station, TX, USA).

## Results

### Pilot study examining agreement between different CA IX stainings

**Figure 1:**
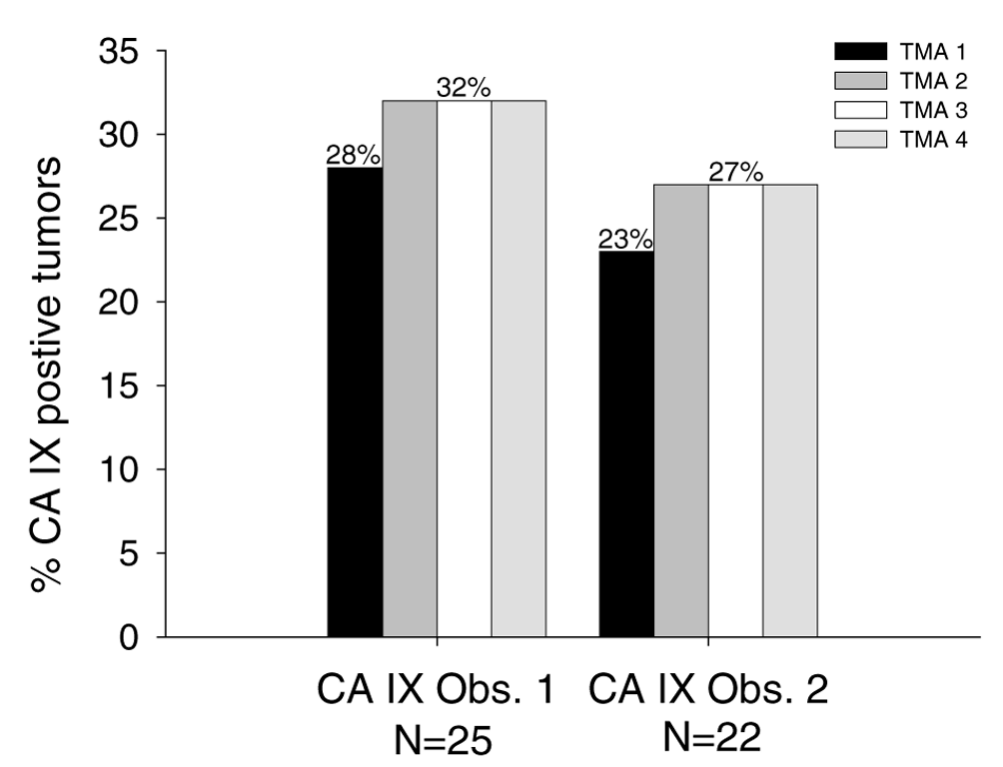
Immunohistochemical analyses of CA IX on TMAs. The tissue microarrays (TMAs) were scored by two observers: observer 1 (Obs. 1) and observer 2 (Obs. 2). Only tumours that were interpretable on all four cores were included in this analysis. The left-most bar in each group (TMA 1) gives the frequency of tumour positivity in one TMA core. The subsequent bars (moving from left to right) show the tumour positivity observed after adding information from addtional cores.

The κ values varied when cut-points determining CA IX tumour positivity was changed (Table 1). Comparing a 1 mm TMA core with the corresponding whole section, a high level of agreement was found using a cut-point of at least 10% of invasive tumour staining. In addition, a high level of agreement was found using central biopsies as compared with peripherally biopsies, both comparing TMA cores with whole sections and comparing TMA cores from two different paraffin blocks from the same tumour. In fact, κ values were of almost the same magnitude using central cores and a cut-point of 10%, as found when comparing stainings of two different whole sections from the same tumour (observer 1: κ = 0.71 versus κ = 0.72; and observer 2: κ = 0.65 versus κ = 0.74). However, caution should be exercised when comparing κ values because they are dependent on distribution, and a slightly higher frequency of CA IX positive tumors was found in the whole sections (28%) as compared with the TMA cores (24%) at a cut-point of 10%. Nevertheless, tables showed the same percentage of discordant cases when comparing CA IX stainings of TMA sections with stainings of corresponding whole sections, and when comparing two whole sections (observer 1: 6/54 versus 3/27; observer 2: 6/54 versus 3/27; tables not presented). Adding positive CA IX information from one extra core to the CA IX information already obtained was shown only to increase the percentage of CA IX positive tumours slightly from 28% to 32% for observer 1 and from 23% to 27% for observer 2 (Figure 1). Adding information from two or three extra cores did not further increase the frequency of CA IX positive tumors. A high interobserver agreement was found both scoring TMA cores and scoring whole sections, with κ values exceeding 0.80 (data not presented).

**Table 1:** Kappa values

	One cell		≥ 10% invasive tumour staining		≥ 20% invasive tumour staining	
	
	Obs. 1	Obs. 2	Obs. 1	Obs. 2	Obs. 1	Obs. 2
TMA biopsy versus whole section (central)	0.56	0.51	0.71	0.65	0.68	0.61
TMA biopsy versus whole section (peripheral)	0.52	0.37	0.59	0.59	0.59	0.58
Two different paraffin blocks (central biopsies)	0.72	0.56	0.69	0.86	0.89	0.86
Two different paraffin blocks (peripheral biopsies)	0.66	0.33	0.42	0.49	0.41	-0.07
Whole sections (two different paraffin blocks)	0.70	0.55	0.72	0.74	0.70	0.70

Presented are κ values that describe comparisons between immunohistochemial stainings for carbonic anhydrase IX of 108 tissue microarray biopsies and 54 whole sections from 27 breast carcinomas. The κ values are presented for two observers and using different cut-points for determining positivity.

### DBCG82 b and c studies

The subgroup of 1,000 patients was well distributed between the two randomization arms for all classical clinicopathological parameters, including CA IX (Table 2).

**Table 2:** Distribution of clinicopathological parameters in 1,000 high-risk breast cancer patients

		Radiotherapy	No radiotherapy	χ^2^
All		489 (49%)	511 (51%)	
Protocol	Pre (DBCG82b)	267 (54%)	296 (58%)	0.3
	Post (DBCG82c)	222 (46%)	215 (42%)	
Tumour size (mm)	<21	171 (35%)	190 (37%)	0.1
	21 to 50	259 (53%)	242 (47%)	
	>50	59 (12%)	79 (15%)	
Positive nodes	None	29 (6%)	31 (6%)	0.8
	1 to 3	222 (46%)	220 (43%)	
	>3	238 (49%)	260 (51%)	
Histopathology	Ductal	428 (88%)	427 (84%)	0.1
	Nonductal	61 (12%)	84 (16%)	
Malignancy grade (ductal only)	Grade 1	98 (23%)	97 (23%)	0.7
	Grade 2	213 (50%)	223 (52%)	
	Grade 3	117 (27%)	107 (25%)	
ER	Positive	327 (67%)	344 (67%)	0.4
	Negative	160 (33%)	167 (33%)	
	Unknown	2 (0%)	0 (0%)	
PgR	Positive	283 (58%)	313 (61%)	0.3
	Negative	205 (42%)	198 (39%)	
	Unknown	1 (0%)	0 (0%)	
HER2	Negative	376 (77%)	404 (79%)	0.6
	Positive	110 (22%)	106 (21%)	
	Unknown	3 (1%)	1 (0%)	
CA IX	Negative	386 (79%)	408 (80%)	0.3
	Positive	80 (16%)	71 (14%)	
	Unknown	23 (5%)	32 (6%)	

Shown is the distribution of various clinicopathological parameters, including oestrogen receptor (ER), progesterone receptor (PgR), human epideraml growth factor receptor (HER)2 and carbonic anhydrase (CA) IX among 1,000 high-risk breast cancer patients randomly assigned to receive or not receive postmastectomy radiotherapy.

### Frequency of CA IX

Immunohistochemical staining for CA IX was performed successfully for 945 out of the 1,000 patients, applying a threshold of at least 10 invasive tumour cells needed to score the slide. A total of 151 out of 945 patients (16%) were classified as being CA IX positive using a cut-point of ≥ 10% invasive tumour staining for CA IX. Applying cut-points of at least one tumour cell, and ≥ 20% and ≥ 30% invasive tumor staining for CA IX, the percentages of CA IX positive tumours were 26%, 13% and 10%, respectively.

### Prognostic value of CA IX

Positive CA IX staining was significantly associated with other markers of poor prognosis such as grade 3 malignant tumours, hormone receptor negative tumours and HER2 positive tumours, although not with positive lymph nodes or large tumour size (Table 3).

**Table 3:** Distribution of clinicopathological parameters: CA IX positive versus CA IX negative high-risk breast cancer patients

		CA IX positive	CA IX negative	χ^2^/*P *value
All		151 (16%)	794 (84%)	
Protocol	Pre (DBCG82b)	89 (59%)	447 (56%)	0.5
	Post (DBCG82c)	62 (41%)	347 (44%)	
Tumour size (mm)	<21	48 (32%)	289 (36%)	0.5
	21 to 50	81 (54%)	393 (49%)	
	>50	22 (15%)	112 (14%)	
Positive nodes	None	6 (3%)	50 (6%)	0.5
	1 to 3	70 (46%)	346 (44%)	
	>3	75 (50%)	398 (50%)	
Histopathology	Ductal	133 (88%)	680 (86%)	0.4
	Nonductal	18 (12%)	114 (14%)	
Malignancy grade	Grade 1	18 (12%)	166 (21%)	*P *< 0.0001
	Grade 2	56 (37%)	357 (45%)	
	Grade 3	59 (39%)	157 (20%)	
	Nonductal	18 (12%)	114 (14%)	
ER	Positive	50 (33%)	581 (73%)	*P *< 0.0001
	Negative	101 (67%)	213 (27%)	
PgR	Positive	47 (31%)	506 (64%)	*P *< 0.0001
	Negative	104 (69%)	288 (36%)	
HER2	Negative	101 (33%)	636 (80%)	*P *< 0.0001
	Positive	50 (67%)	636 (80%)	

Shown is the distribution of various clinicopathological parameters among carbonic anhydrase (CA) IX positive and CA IX negative high-risk breast cancer patients randomized to receive or not receive postmastectomy radiotherapy. ER, oestrogen receptor; HER, human epidermal growth factor; PgR, progesterone receptor.

In univariate analyses of the 945 patients with assessable CA IX stainings, positive CA IX status was significantly associated with increased overall mortality (HR 1.30, 95% confidence interval [CI] 1.06 to 1.60) and disease-specific mortality (HR 1.29, 95% CI 1.02 to 1.62), but not with increased probability of DM (HR 1.23, 95% CI 0.98 to 1.54) or increased probability of LRR (HR 1.28, 95% CI 0.82 to 2.01). However, the significance of the associations of positive CA IX staining with overall and disease-specific mortality disappeared in multivariate analysis (*P *= 0.2), after correction for positive lymph nodes, tumour size, grade of malignancy, hormone receptor and HER2 receptor status, menopausal status/systemic treatment, and randomization, which were all independent prognostic markers. Excluding hormonal receptor status from the multivariate analysis did not change this finding.

Multivariate subgroup analyses were performed for all subgroups of patients presented in Table 3. Positive CA IX was significantly associated with increased overall mortality only in the subgroups of postmenopausal women (*P *= 0.005), women with one to three positive lymph nodes (*P *= 0.04), women with ER-positive tumours (*P *= 0.003) and women with PgR-positive tumours (*P *= 0.03). To compare our results with those reported by Brennan and coworkers [8], we conducted further subgroup analyses of the premenopausal women. Those analyses showed that CA IX was not an independent prognostic marker of overall mortality among premenopausal women with one to three positive lymph nodes (*P *= 0.3), but it was among premenopausal women with more than three positive lymph nodes (*P *= 0.05). CA IX has been suggested to be an intervening variable in ER downregulation, which was why it was excluded from multivariate analyses by Brennan and coworkers [8]. However, when hormone receptor status was excluded from the multivariate analysis of premenopausal women with more than three positive lymph nodes, the statistical significance of the finding disappeared (*P *= 0.4). Interestingly, in the subgroup of postmenopausal women (who were additionally treated with tamoxifen), CA IX was a stronger prognostic marker of overall mortality, as was hormone receptor status. Performing further subgroup analyses of the postmenopausal women showed that when this group was further separated according to randomization status, CA IX was significantly associated with overall mortality only within the subgroup of women randomly assigned to radiotherapy (*P *= 0.03) and it was a stronger prognostic marker of overall mortality, as was hormonal receptor status (*P *= 0.3). Finally, a subgroup analysis of hormone receptor positive women revealed that CA IX was an independent prognostic marker of OS only in the subgroup of postmenopausal women.

Changing the cut-points for determining CA IX positivity to just one tumour cell, or ≥ 20% or ≥ 30% invasive tumour staining did not improve the prognostic value of positive CA IX staining in the total group of 945 patients.

### CA IX and response to PMRT

**Figure 2:**
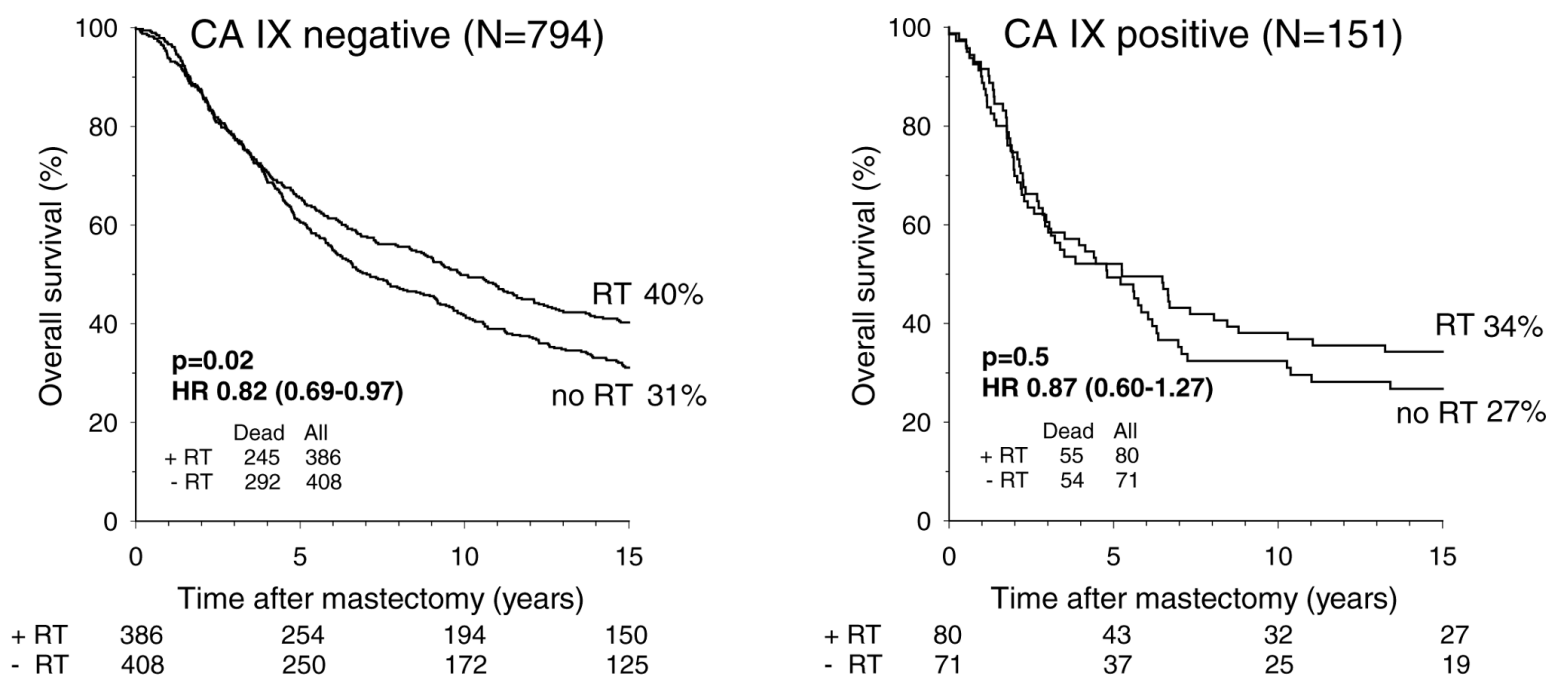
Overall survival in high-risk breast cancer patients. Shown are Kaplan-Meier probability plots of overall survival in high-risk breast cancer patients as a function of randomization to postmastectomy radiotherapy (RT) within the subgroups of carbonic anhydrase (CA) IX negative and CA IX positive patients. The values given in parentheses after hazard ratios (HRs) are the 95% confidence intervals.

Kaplan-Meier probability plots of OS after PMRT among CA IX positive and CA IX negative patients revealed improved survival after PMRT in both subgroups of patients (Figure 2). Fifteen-year OS probabilities were improved by 7% and 9% after PMRT for the subgroups of CA IX positive and CA IX negative patients, respectively. The HR of overall mortality after PMRT was 0.87 (95% CI 0.60 to 1.27) for the small subgroup of 151 CA IX positive patients and 0.82 (95% CI 0.69 to 0.97) for the large subgroup of 813 CA IX negative patients. HRs and 95% CIs after PMRT did not differ significantly between CA IX positive and CA IX negative patients for OS, DSS, DM, or LRR (Table 4). In multivariate analysis by Cox regression, no significant interaction was found between CA IX and randomization status for any of the four end-points. Similar tendencies were observed when the women were separated into premenopausal and postmenopausal women, and into women with one to three positive nodes and those with more than three positive nodes.

**Table 4:** Hazard ratios

	CA IX positive (n = 151)	CA IX negative (n = 794)
Overall mortality	0.87 (0.60–1.27)	0.82 (0.69–0.97)
Disease-specific mortality	0.83 (0.55–1.27)	0.76 (0.63–0.93)
Distant metastases	0.83 (0.54–1.25)	0.76 (0.63–0.91)
Locoregional recurrence	0.23 (0.08–0.61)	0.16 (0.09–0.28)

Presented are hazard ratios (95% confidence intervals) of overall mortality, disease-specific mortality, distant metastases and loco-regional recurrence probabilities after postmastectomy radiotherapy in carbonic anhydrase (CA) IX positive and in CA IX negative high-risk breast cancer patients.

Changing the cutpoints to at least one tumour cell, or ≥ 20% or ≥ 30% invasive tumour staining did not improve the predictive value of positive CA IX staining (data not presented).

## Discussion

None of the three hypotheses was verified within this 945-patient subgroup in which CA IX staining was successfully applied. CA IX was not an independent prognostic marker of OS, DSS, DM, or LRR when analyzed in the total group of 945 patients or when analyzed in any of the two randomization arms. Furthermore, no significantly smaller reduction in survival or LRR after PMRT was found in CA IX positive patients as compared with CA IX negative patients.

The subgroup of 1,000 patients was representative of the total cohort of 3,083 patients, with patients being well distributed between the two randomization arms and producing similar outcome data as observed among the total 3,083 patients [23]. Hence, the likelihood that the findings are due to selection bias is minimal.

It appears that CA IX is only a weak, nonindependent prognostic marker, because correction for apparently stronger prognostic markers such as positive nodes, tumour size, malignancy grade, hormone receptor status, HER2 status, menopausal status/systemic treatment and randomization eliminated the effect of CA IX status. Previously, positive CA IX staining was found to be an independent prognostic marker of poor survival in breast cancer in just a few studies [7,8,13,14]. Brennan and coworkers [8] found the prognostic value of CA IX among premenopausal women to be restricted to those women treated with tamoxifen and, among women who were not treated with tamoxifen, to those with one to three positive lymph nodes. In disagreement with those findings, we found CA IX not to be an independent prognostic marker of OS within the subgroup of premenopausal women, either in the whole subgroup or in those with one to three positive lymph nodes. However, we found CA IX to be an independent prognostic marker for OS in the subgroup of premenopausal women with more than three positive lymph nodes. However, the statistical significance disappeared if hormonal receptor status was excluded from the multivariate regression analysis, as was the case in the analyses conducted by Brennan and coworkers. Excluding hormone receptor from the analyses, could be justified by CA IX being an intervening variable in ER down-regulation. In accordance herewith, hypoxia and ER downregulation have been linked in some studies [24-26]. Furthermore, in accordance with the findings presented by Brennan and coworkers, we found CA IX positivity to be significantly associated with ER and PgR negative status in 2 × 2 tables; it was also significantly associated with OS in multivariate analyses within the subgroup of postmenopausal women (all treated with tamoxifen), and within the total subgroup of ER-positive patients and the subgroup of ER-positive postmenopausal women treated with tamoxifen. Indeed, CA IX was a stronger prognostic marker for OS among postmenopausal women as was hormone receptor status.

Hence, CA IX may be a predictor of response to tamoxifen. However, the predictive value of CA IX for response to tamoxifen treatment was previously examined and no evidence of this was found [8]. In addition, no evidence was found linking hypoxia-inducible factor-1 with response to tamoxifen [27]. Performing further subgroup analyses, we found that the independent prognostic value of CA IX for OS was restricted to postmenopausal women randomly assigned to radiotherapy, which suggests that CA IX status may predict response to radiotherapy. However, in the present study we found no evidence of an association between CA IX and response to radiotherapy, either in terms of survival or LRR. Kaplan-Meier probability plots showed an improved OS after PMRT within the two subgroups of CA IX negative and CA IX positive women (15-year OS probabilities improved by 9% and 7%, respectively). The HR was significant only in the large subgroup of patients who were 813 CA IX negative, but this is to be expected because an anticipated improvement in survival of about 8% to 10% would not be expected to be statistically significant in a subgroup of only 151 CA IX positive patients.

To summarize, that CA IX may have potential prognostic value for survival among postmenopausal women treated with radiotherapy and tamoxifen cannot be excluded. It should be kept in mind, however, that we did not find CA IX to be an independent prognostic marker within the large series of 945 patients or within either of the two randomization arms. Caution should therefore be exercised when interpreting results obtained from extended subgroup analyses.

The fact that CA IX did not predict outcome after PMRT within this series of 945 patients is in disagreement with other work [7], which suggested a reduction in survival after PMRT that was restricted to CA IX positive patients. However, that study included TMAs from only 160 patients and defined tumour positivity as staining of at least one invasive tumour cell in a section, which we have demonstrated may have an inferior reproducibility as compared with using a cut-point of ≥ 10%. Brennan and coworkers [8] suggested that positive CA IX might be a marker of resistance to radiotherapy among patients with one to three positive lymph nodes. We found no evidence of this, with similar reductions in OS after PMRT for CA IX negative and CA IX positive patients with one to three positive lymph nodes. Our results indicate that CA IX alone is not a specific marker of resistance to radiotherapy and may be the presence of hypoxic tumour cells, which is in accordance with other reports from our laboratory. *In vitro *studies have shown that increasing levels of hypoxia induced a rise in CA IX expression, which was clearly pH dependent. CA IX increased in response to decreasing oxygen tension at pH above 6.5 but, despite decreasing oxygen tension, did not increase in the most acidic range measured in tumours (pH 6.3 to 6.5); this suggests that the relation between CA IX and hypoxia is more complicated. Another study examining 57 head and neck squamous cell carcinomas found no correlation between immunohistochemically determined CA IX status and tumour oxygenation status using partial oxygen tension needle electrodes [28]. Finally, a recent study [9] revealed that CA IX, identified using immunohistochemistry, did not predict response to nimorazole (a hypoxic sensitizer) in a series of 320 patients with head and neck squamous cell carcinomas.

However, it should be borne in mind that using TMAs for immunohistochemical examination of CA IX underestimates the frequency of CA IX positive tumours. Invasive tumor cells do not express CA IX diffusely but rather focally and more frequently in tumor cells near necrotic areas [12,13,22,29]. Frequencies of CA IX positive tumours of varying magnitude have been reported in studies examining whole sections (46% to 48%) [10,13] and TMAs (18% to 28%) [7,11], and the same tendency was found in our pilot study examining agreement between CA IX stainings, albeit not as marked. In addition, we found that adding one extra TMA biopsy slightly increased the frequency of CA IX positive tumors but adding two or three extra TMA biopsies did not further increase the CA IX frequency. However, this contrasts with findings reported by Goethals and coworkers [30], who applied staining for CA IX to colon cancer. They concluded that in order to reduce the number of CA IX negative cases, four or five punches from each tumour was apparently optimal. We opted to use central cores in the DBCG82 study rather than peripheral cores, based on the findings of the pilot study. This could be a point of criticism because in our study we did not find strictly the same amount of invasive tumour cells in central and peripheral biopsies; also, the percentages of assessable tumour cells in each core differed between different tumour blocks, because of differences in size of the invasive areas. However, in general the original tumours were large, because they were selected only if at least three paraffin-embedded tumour blocks containing verified invasive tumour could be located. For these reasons, this shortcoming of our study probably has little impact on our findings. An alternative is to use whole sections instead of TMAs. However, the reproducibility of findings in whole sections from two different paraffin blocks from the same tumour was not notably better than comparing a central TMA biopsy with the corresponding whole section in our study. It has been argued that a whole section is still only a small fraction of the entire tumour, and when one looks at something rare and unequally distributed, one might derive the most accurate picture of that distribution by looking at a small proportion of tissue such as a TMA biopsy. In addition, it must be stressed that the set up in our pilot study (as well as in the study by Goethals and colleagues [30]) was not designed to evaluate the accuracy of TMAs, because these were not compared with the 'truth' but were compared with whole sections, which account for only part of the truth.

## Conclusion

This is the largest study of CA IX to date; in 945 high-risk breast cancer patients randomly assigned to receive PMRT plus systemic therapy or systemic therapy alone, we found CA IX to be significantly associated with OS and DSS but not with DM or LRR in univariate analyses. CA IX was not an independent prognostic marker for any of the end-points analyzed. However, we cannot rule out that CA IX may be an independent prognostic marker for survival among different subgroups of women. Among other findings, we identified a potential association between CA IX and response to tamoxifen in postmenopausal women treated with tamoxifen and PMRT and in the subgroup of hormone receptor positive women. CA IX was not associated with response to PMRT. Fifteen-year OS probabilities were improved by 9% and 7% after PMRT for the subgroups of CA IX negative and CA IX positive patients, respectively. The improvement in OS after PMRT did not reach statistical significance in the small subgroup of 151 CA IX positive patients.

## Abbreviations

CA = carbonic anhydrase; CMF = cyclophosfamide, methotrexate and fluorouracil; CI = confidence interval; DBCG82 = Danish Breast Cancer Cooperative Group; DM = distant metastases; DSS = disease-specific survival; ER = oestrogen receptor; HER = human epidermal growth factor; HR = hazard ratio; LRR = locoregional recurrence; OS = overall survival; PgR = progesterone receptor; PMRT = postmastectomy radiotherapy; TMA = tissue microarray.

## Competing interests

The authors declare that they have no competing interests.

## Authors' contributions

MO and JO were responsible for data collection in the original clinical study. Recent follow up of clinical data was performed by HMN, MO and JO. MK and JO were conceived and designed the biological study. HK and MK collected paraffin-embedded tumour blocks. MK constructed TMAs. MK, JA and FBS were responsible for staining of TMA sections. MK and FBS scored TMA sections. MK and JO analyzed and interpreted the data. MK wrote the manuscript. All authors read and approved the manuscript.

The corresponding author (MK) had full access to all of the data used in the study and has final responsibility for the decision to submit the paper.
